# Lysine attenuates acute lung injury by restoring α-tubulin acetylation and ciliary activity

**DOI:** 10.1038/s41420-026-03025-x

**Published:** 2026-03-16

**Authors:** Wenyu Yang, Xiaoxiao Meng, Yong Zhu, Xinrun Ma, Zhuoran Cao, Mengmeng Wang, Wentao Dai, Yiming Tao, Xiangdong Jian, Rui Tian, Zhengfeng Yang, Ruilan Wang

**Affiliations:** 1 Department of Critical Care Medicine, Shanghai General Hospital, Shanghai Jiaotong University, School of Medicine, Shanghai, China; 2 Precision Research Center for Refractory Diseases, Shanghai Jiao Tong University Pioneer Research Institute for Molecular and Cell Therapies, Shanghai General Hospital, Shanghai Jiao Tong University School of Medicine, Shanghai, China; 3 State Key Laboratory of Innovative Immunotherapy, School of Pharmaceutical Sciences, Shanghai Jiao Tong University, Shanghai, China; 4 Shanghai-MOST Key Laboratory of Health and Disease Genomics & NHC Key Laboratory of Reproduction Regulation, Shanghai Institute for Biomedical and Pharmaceutical Technologies (SIBPT), Fudan University, Shanghai, China; 5 Department of Poisoning and Occupational Diseases, Emergency, Qilu Hospital, Cheeloo College of Medicine, Shandong University, Jinan, Shandong China

**Keywords:** Respiratory distress syndrome, Calcium signalling, Calcium channels, Cilia, Cadherins

## Abstract

Acute respiratory distress syndrome and pulmonary fibrosis stemming from severe acute lung injury (ALI) continue to incur high mortality due to ineffective pulmonary regeneration. While metabolic reprogramming is known to support alveolar epithelial repair, the specific role of amino acid metabolism remains enigmatic. Through integration of scRNA-seq mining analysis of human ALI samples and targeted plasma metabolomics, we identified that lysine was largely declined in injured pulmonary epithelium, accompanied by a deficiency of mitochondrial metabolism. Lysine supplementation dramatically improved survival (from 0% to 62.5% in mice), attenuated extracellular matrix deposition and alveolitis, and suppressed inflammation in murine and non-human primate ALI models. Mechanistically, lysine replenished acetyl-CoA to restore α-tubulin acetylation for rescuing ciliary TRPC1 localization, which prevented pathological STIM1-TRPC1 complex formation, thereby blocking calcium influx-reduced E-Cadherin/ZO-1 abundance in pulmonary epithelial cells. Notably, ciliogenesis preferentially occurred in SFTPC+ alveolar epithelial type II (AT2) cells; thus, lysine supplementation would promote regenerative activation of AT2 cells. Our work established lysine as a metabolic-structural orchestrator that coordinates acetyl-CoA availability to calcium homeostasis and epithelial repair through tubulin-mediated ciliary signaling.

## Introduction

Acute lung injury (ALI) is a critical illness syndrome triggered by multiple insults, including severe infections such as COVID-19/Influenza A, radiation, chemotherapy, toxins, chronic obstructive pulmonary disease (COPD), etc [1–4]. Inadequate management makes ALI swiftly progress into acute respiratory distress syndrome (ARDS) and pulmonary fibrosis (PF), resulting in respiratory failure followed by multiple organ failure and high mortality [5]. The incidence of ARDS is around 10% globally, with a 35–45% in-hospital case fatality rate, especially those who suffered PF progression [6]. Pharmacologic therapeutic strategies, including anti-inflammatory treatments, present transient improvements; however, they do not reduce short-term or long-term mortality. Supportive therapies, including lung-protective ventilation, prone positioning, fluid management, and even extracorporeal membrane oxygenation, are approaches typically applied in the clinic with promising efficacy in some patients [5, 7, 8]. These observations indicated the importance of endogenous regenerative mechanisms facilitated by an elegant buffer environment in alleviating ALI. In line with this assumption, recent studies have revealed the stemness of pulmonary alveolar epithelial type II cells (AT 2) to differentiation into pulmonary alveolar epithelial type I cells (AT 1) for pulmonary regeneration from lung injury [9]. Mild injury of pulmonary epithelial cells leads to inflammation for replacing the damaged epithelial or endothelial cells to maintain barrier function and integrity, from which inflammatory monocytes and neutrophils were recruited with cytokine production, facilitating epithelial-mesenchymal transition (EMT) in alveolar epithelial cells for tissue repair [10]. However, severe or persistent damage exacerbates the differentiation of alveolar epithelium into pro-fibrotic mesenchymal states but not AT 1 cell, which aggravates fibrosis by synthesizing excessive amounts of extracellular matrix components, leading to a poor prognosis of ALI [11, 12].

Pulmonary alveolar epithelial cells are thus critical for lung repair during ALI, with several growth factors identified to promote the proliferation of alveolar epithelium, including AT 2 cell; however, they have not yet been applied in clinical management for ALI [5]. Considering cell proliferation requires an amount of energy from cellular metabolism [13], a sole treatment of growth factors would reach limited efficacy for alveolar epithelium activation during ALI. Emerging evidence showed abnormal metabolism in pulmonary diseases, including reduced lipid metabolism during COPD progression [14], increased lipid biosynthesis for maintaining AT 2 activities in response to smoking-relevant COPD [15], and palmitate utilization compensated for the impaired glucose and mitochondrial metabolism in AT 2 cells after the short-term cigarette smoke exposure [16]. These adaptations reflect a complex interplay between glucose-lipid metabolic crosstalk during mild injury triggered by smoking. In line with these observations, severe ALI progression into PF raised a much-complicated dysregulation of lipid, glucose, and amino acid metabolism in AT 2 cells [17]. Except for the compensation for glucose metabolism or from the metabolic crosstalk between glucose and lipid, little is known about amino acid metabolism in response to the dysregulated metabolic network. Several studies, including ours, identified the importance of glutamine both for compensation of impaired mitochondrial metabolism to protect the lung epithelium from injury [18] and fibroblast-to-myofibroblast transition (FMT) process facilitating PF progression [19, 20], two contradictory aspects for the prognosis of ALI. As amino acid metabolism serves as a compensatory mechanism post glucose/lipid pathway failure [21], it is important to elucidate specific amino acid functions for modulating the activity of alveolar epithelium, especially AT 2, in severe ALI.

Here, we utilized a paraquat (PQ) poisoning-induced severe ALI model that presents irreversible PF progression in a short period to identify the potential importance of amino acid metabolism in pulmonary alveolar epithelial cells for lung regeneration and repair. Our study confirmed the impairment of mitochondrial metabolism during severe ALI progression and further identified the potency of lysine for replenishing the acetyl-CoA pool in maintaining the cilia activity and E-Cadherin integrity in pulmonary epithelial cells, uncovering a novel metabolic regulatory axis connecting lysine supplementation with calcium homeostasis in ALI. As lysine has already been applied in the clinic, lysine could thus be a potential safe adjunct strategy to treat patients suffering from ALI.

## Results

### Severe ALI leads to metabolic rewiring in pulmonary alveolar epithelial cells

**Fig. 1:**
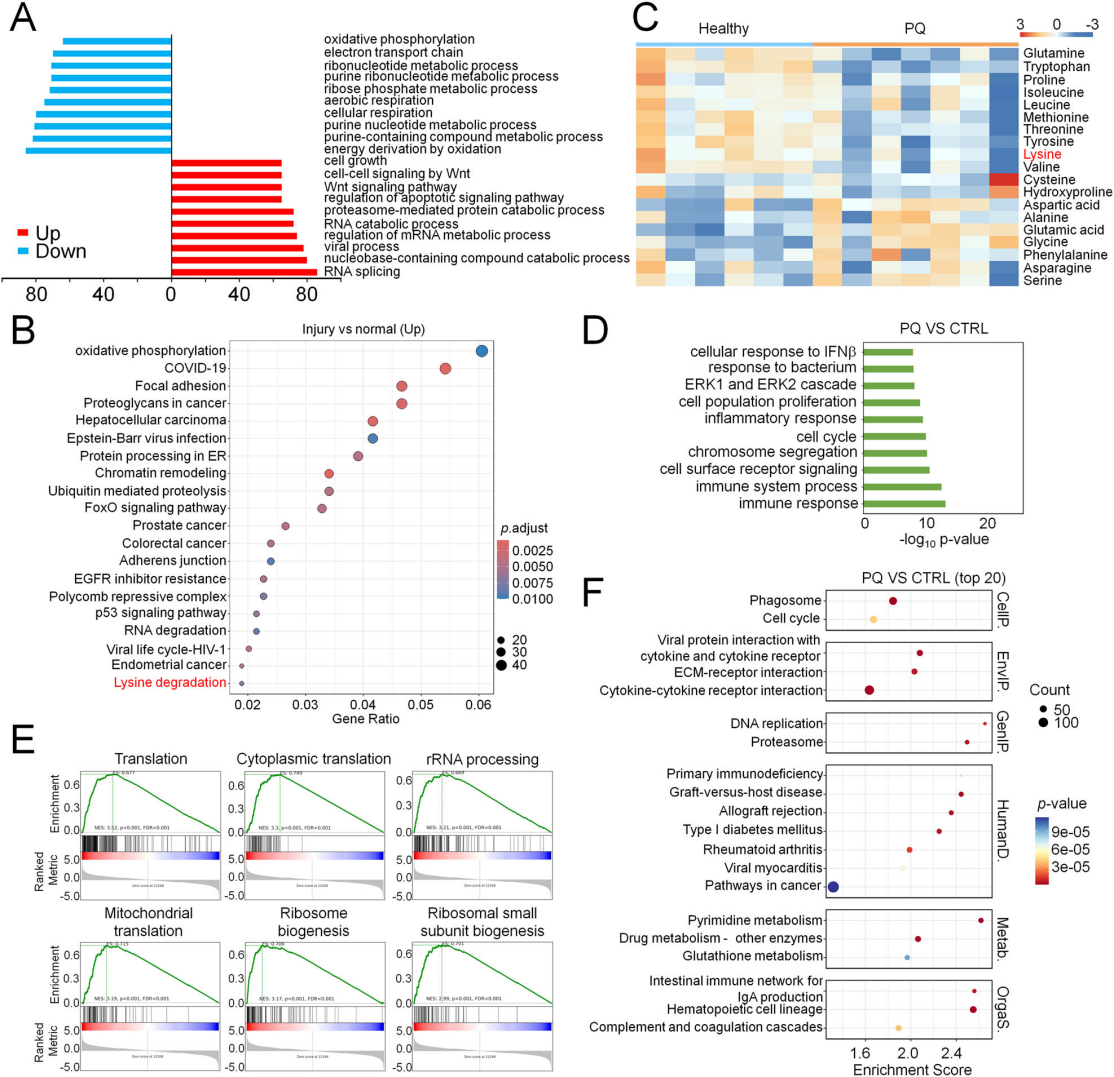
Severe ALI leads to metabolic rewiring in pulmonary alveolar epithelial cells. GO enrichment (**A**) and KEGG analysis (**B**) showing top-ranked biological processes and KEGG pathways in pulmonary epithelial cells between normal and PQ-poisoned patients by scRNA seq mining analysis of published GEO database (GSE 132771 and GSE 231647). **C** Amino acid metabolic profiles measurement in plasma from healthy volunteers (*n* = 6) or PQ-poisoned patients (*n* = 7). Go enrichment (**D**) GSEA (**E**) and KEGG (**F**) analysis showing top-ranked biological processes and pathways in lung tissues injured with or without PQ.

scRNA-seq mining analysis of PQ-poisoned patients suffering severe ALI identified pulmonary alveolar epithelial cells as the most significantly dysregulated population, exhibiting concurrent suppression of mitochondrial metabolism and activation of RNA metabolism (Fig. 1A). Interestingly, KEGG analysis revealed enrichment of oxidative phosphorylation and RNA degradation in injured lung tissues (Fig. 1B). These observations indicated that mitochondrial metabolism was highly activated with exhaustion for alveolar epithelium recovery during ALI progression. Notably, the lysine degradation pathway was upregulated in injured alveolar epithelial cells (Fig. 1B). Specific genes relevant to lysine degradation included several histone methyltransferases and metabolic enzymes for lysine catabolism (DHTKD1 and ALDH7A1). DHTKD1 and ALDH7A1 are essential for lysine metabolic conversion to acetyl-CoA [22], therefore correlating with marked serum lysine decline in patients (Fig. 1C). An observation of essential amino acids except phenylalanine all decreased further indicated the accelerated utilization approaching exhaustion in response to mitochondrial bioenergetic crisis in PQ-poisoned patients. The murine ALI model recapitulated this metabolic rewiring, including activated signals for cell growth, ribosome biogenesis, cell cycle and DNA replication, accompanied by immune responses (Fig. 1D–F), reflecting heightened biosynthetic and proliferative demands for pulmonary tissue repair. Collectively, these adaptive responses indicated that amino acid metabolism, particularly lysine catabolism, was a salvage pathway to compensate for collapsed glucose/fatty acid oxidation due to severe ALI, redirecting metabolic flux to sustain alveolar epithelial viability during an energy crisis.

### Lysine largely suppresses PQ-induced ALI progression

**Fig. 2:**
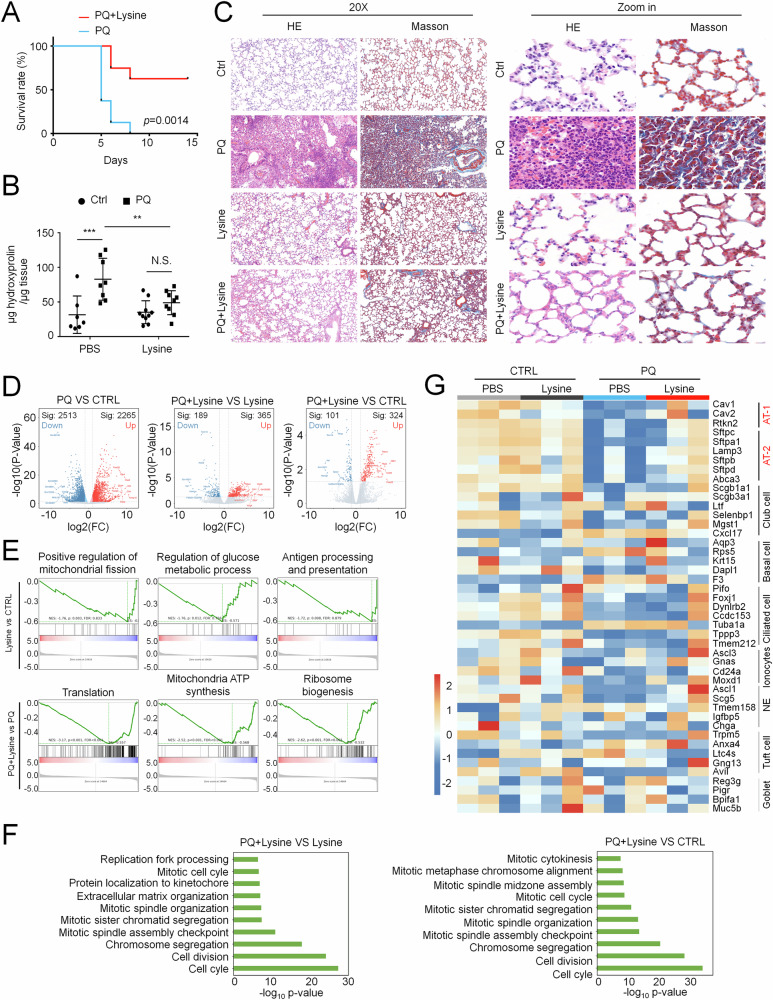
Lysine alleviates PQ-induced acute lung injury. **A** Kaplan–Meier analysis of survival rate in PQ-poisoned mice treated w/wo lysine. *n* = 8 initially in each group. **B** ELISA analysis of hydroxyproline in PQ-poisoned lungs treated w/wo lysine for 3 days. Mean ± SD, **P < 0.01, ***P < 0.001; Two-Way ANOVA. NS, not significant. **C** HE staining and Masson staining of PQ-poisoned lungs treated w/wo lysine for 3 days. Images are representative of more than three mice in each group. **D** Volcano plots showing gene expression profiles in PQ-injured lung tissues treated w/wo lysine for 3 days. **E** GSEA analysis presents different top-ranked biological processes between CTRL and PQ-injured lung tissues in the condition with lysine supplementation. **F** GO enrichment revealed top-ranked biological processes in PQ-injured lung tissues with lysine supplementation compared to either lysine-treated or untreated lung tissues. **G** Heatmap showing marker genes expression of pulmonary epithelial cell subsets in PQ-injured lung tissues w/wo lysine supplementation.

Considering the observations of both the reduction of serum lysine in patients and enhanced lysine degradation pathways in pulmonary epithelial cells, in this study, we focused on the therapeutic potential of lysine in ALI. Previous work reported therapeutic benefits of lysine acetylsalicylate, a hydrolysable aspirin prodrug, in PQ-intoxicated rats [23]. While that study primarily attributed efficacy to aspirin’s anti-inflammatory properties, it concurrently indicated lysine’s therapeutic potential, given in vivo hydrolysis into active components. We then evaluated lysine supplementation in the PQ-induced ALI model. Administration of lysine in PQ-poisoned mice largely increased the survival rate, as all PQ-treated mice all died at day 7, whereas 62.5% mice survived with lysine supplementation (Fig. 2A). The examination of hydroxyproline levels in the lung revealed that PQ-induced ALI, accompanied by extracellular matrix deposition were significantly alleviated by co-administration with lysine (Fig. 2B). The HE and Masson staining further confirmed that ALI-driven alveolitis was almost healed in lysine treated group, accompanied by recovered food intake and water intake (Figs. 2C and S1A–D). To further understand the recovery process of ALI by lysine treatment, we performed RNA-seq analysis on the lungs of mice. Addition of lysine largely normalized the drastic alteration of gene expression profiles during ALI (Fig. 2D), confirming the therapeutic efficacy of lysine. GSEA analysis revealed a context-dependent modulation by lysine supplementation. Particularly, lysine treatment downregulated only mitochondrial activity in the physiological condition, while during ALI, it reduced both ribosome-relevant protein synthesis and mitochondrial metabolism (Fig. 2E). The different responses suggested that lysine administration was available for functional compensation of mitochondrial metabolism, which could further redirect resources to epithelium repair during ALI. Indeed, lysine supplementation in injured mice present significantly different biological processes relevant to cell growth, compared to either lysine treated- or PBS- treated mice (Fig. 2F). A further detailed analysis of marker genes for pulmonary epithelial cell subsets identified that AT 2 cells and AT 1 cells were mostly injured during ALI, which could be largely recovered by lysine supplementation (Figs. 2G and S1E, F). Taken together, these results strongly suggested that lysine largely reversed PQ-induced ALI via the regeneration of alveolar epithelium.

### Lysine alleviates inflammatory responses triggered by ALI

**Fig. 3:**
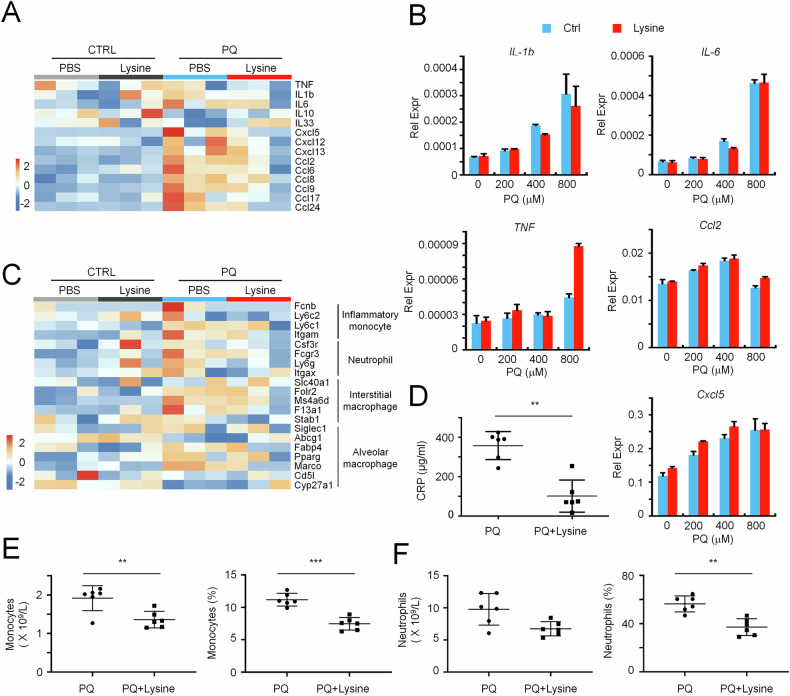
Lysine treatment attenuates PQ-induced immune activation. **A** Heatmap showing cytokine expression profiles in PQ-injured lung tissues w/wo lysine supplementation. **B** QPCR analysis of cytokines abundance in PQ-injured A549 cells w/wo lysine supplementation. **C** Heatmap showing marker genes expression of innate immune cells in PQ-injured lung tissues w/wo lysine supplementation. ELISA analysis of serum C-reactive protein (CRP) (**D**) and the number or percentage of monocytes (**E**) and neutrophils (**F**) analyzed from laboratory examination of blood in PQ-poisoned cynomolgus monkey treated w/wo lysine. Each dot represents the result of measurement at different time points. Mean ± SD., **P < 0.01, ***P < 0.001; unpaired two–tailed Student’s *t* *test*.

Lysine was once considered in clinics for preventing or treating cold sores due to herpes simplex with little side effects [24]. Lysine has also been proven to prevent and treat brain trauma and brain ischemia [25]. Consistently, we observed that the biological process relevant to immune responses was reduced by lysine treatment in the physiological condition. As alveolar epithelial cells have been reported to produce inflammatory cytokines during ALI for disease progression [26], we then analyzed the abundance of cytokines and chemokines. Heatmap analysis revealed multiple relevant genes were largely reduced with lysine supplementation during ALI, especially chemokines for the recruitment of immune cells (Fig. 3A). However, cytokines that can be produced by alveolar epithelial cells were not suppressed by lysine supplementation (Fig. 3B). A further detailed analysis on marker genes of myeloid cell subsets, mainly producing inflammatory cytokines for ALI progression [27], indicated that all these immune cells were largely enriched during ALI and declined with lysine supplementation (Fig. 3C). Similar results were observed by using a PQ-injured cynomolgus model, which the amount of C-reactive protein (CRP) was largely reduced in lysine-treated cynomolgus, accompanied with reduced number and percentage of peripheral monocytes and neutrophils (Fig. 3D–F). As alveolar epithelium damage leads to the recruitment of inflammatory monocytes and neutrophils, followed by inflammatory macrophage activation [10], lysine-reduced inflammatory responses during ALI could be the result of alveolar epithelial recovery, from which lysine did not directly modulate cytokine production in pulmonary epithelial cells.

### Lysine preserves E-Cadherin integrity, epithelial property and ciliary activity during ALI

**Fig. 4:**
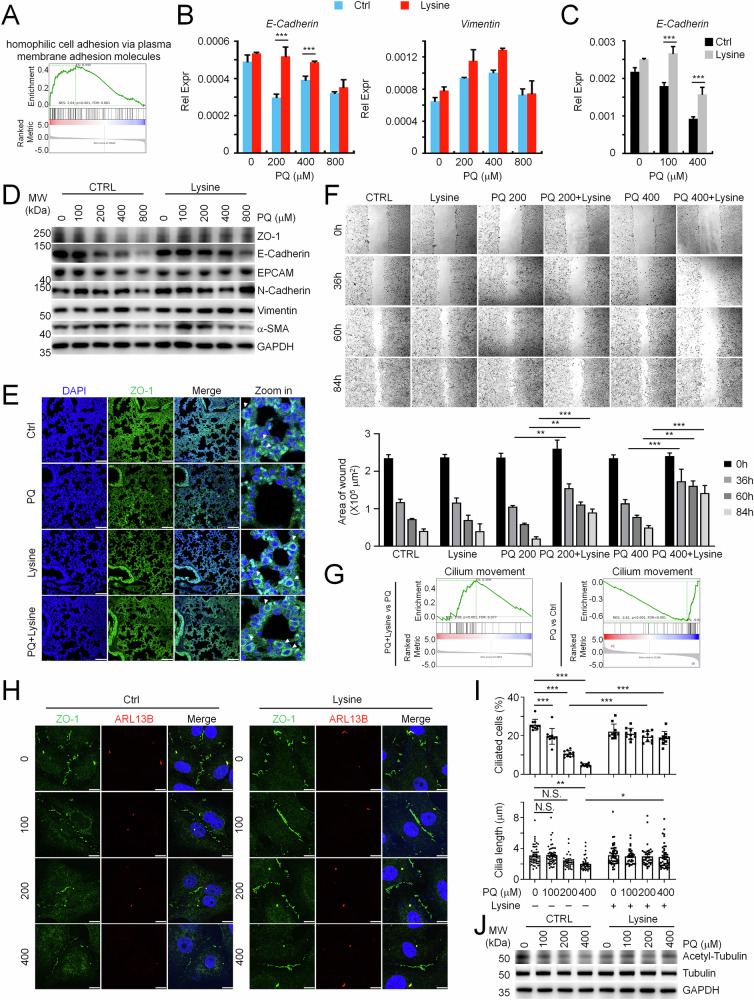
Lysine restored PQ-impaired E-Cadherin integrity, epithelial property and ciliogenesis. **A** GSEA analysis uncovered homophilic cell adhesion via plasma membrane adhesion molecules enhanced in PQ-injured lung tissues with lysine supplementation compared to untreated group. QPCR analysis of E-Cadherin and Vimentin expression in PQ-injured A549 cells (**B**) and MLE-12 cells (**C**) w/wo lysine supplementation (*n* = 3). **D** WB analysis of ZO-1, E-Cadherin, EPCAM, N-Cadherin, Vimentin, and α-SMA expression in PQ-injured A549 cells w/wo lysine supplementation. **E** Immunofluorescence analysis of ZO-1 abundance in PQ-poisoned lungs treated w/wo lysine for 3 days. Scale bar, 100 μm. **F** A549 cells treated w/wo indicated concentration of PQ for 12 h. Wound healing analysis of the injured cells w/wo lysine supplementation for indicated times. **G** GSEA analysis uncovered cilium movement impaired in PQ-injured lung tissues but restored with lysine supplementation. **H** Immunofluorescence analysis of ZO-1 and ARL13B in PQ-injured A549 cells w/wo lysine supplementation. Scale bar, 10 μm. **I** Quantification of percentage of cilia positive cells and cilia length, indicated by ARL13B signal, in PQ-injured A549 cells w/wo lysine supplementation. **J** WB analysis of acetyl-α-Tubulin in PQ-injured A549 cells w/wo lysine supplementation. Mean ± SD., *P < 0.05, **P < 0.01, ***P < 0.001; Two-Way ANOVA. NS, not significant.

We and others both found that PQ-induced ALI is accompanied by EMT for disease progression [28–30]. Our above observations indicated that lysine mainly modulated epithelial properties but not inflammatory cytokine production in alveolar epithelial cells. GSEA analysis did find that lysine treatment recovered homophilic cell adhesion via plasma membrane adhesion molecules, with the majority of enriched genes (96 in total) encoding cadherin superfamily proteins or cadherin-related molecules (Fig. 4A). Therefore, we assumed that lysine would mainly preserve the epithelial property of alveolar epithelial cells for ALI recovery. In line with this assumption, we found that the decreased E-Cadherin, ZO-1 and EPCAM expression by PQ treatment could be largely prevented by additional lysine supplementation, yet the effect on EPCAM was less pronounced. Notably, though N-Cadherin, Vimentin and α-SMA, the mesenchymal markers, could be enhanced with PQ treatment, lysine did not present impressive and consistent normalization on the upregulation of these mesenchymal markers compared to the preservation of epithelial markers (Figs. 4B–D and S2A–C). Consistently, immunofluorescence staining of lung tissues from PQ-injured mice revealed a pronounced loss of ZO-1 in alveolar epithelia, which was substantially recovered upon lysine administration (Fig. 4E). These observations together suggested that lysine mainly preserved E-Cadherin integrity and epithelial property during alveolar epithelial injury. E-Cadherin has been reported to maintain cell adhesion, which limits cell motility but facilitates the well-organized cell arrangement in multiple tissues [31, 32]. We did find a specific decreased movement in injured pulmonary epithelial cells with lysine supplementation (Fig. 4F). Next, we wondered how lysine preserved E-Cadherin integrity and epithelial property. GSEA analysis presents a specific enhancement of cilium movement with lysine supplementation in injured lung tissues. Consistently, cilium movement was impaired during ALI (Fig. 4G). Immunofluorescence analysis further revealed that ZO-1 deficiency was accompanied by a reduced percentage of cilia-positive cells and a slightly but significantly declined cilia length in injured pulmonary epithelial cells (Fig. 4H). Importantly, lysine supplementation restored cilia deficiency in injured cells, with ZO-1 recovered only in cilia-positive populations (Fig. 4G–I). Notably, some signals of ZO-1 also co-localized with ARL13B, indicating that cilia integrity directly preserves epithelial properties. Consistently, we observed a deficiency of α-Tubulin acetylation, the major component for ciliogenesis, with PQ stimulation, which could be almost normalized by lysine supplementation (Figs. 4J and S2E). Notably, airway epithelium harbors ciliated cells equipped with motile cilia essential for mucociliary clearance, distinct from the immotile cilia in alveolar epithelium [33]. While lysine supplementation seems to activate ciliated cells in the physiological condition, it conferred limited rescue during ALI (Fig. 2G), indicating minimal contributions to ALI resolution via ciliated cells modulation. Nevertheless, these observations provided evidence for lysine preserved-cilia activation in maintaining the epithelial property during ALI.

### Lysine facilitates ciliary TRPC1 localization to restrain intracellular calcium increase-mediated E-Cadherin reduction during ALI

**Fig. 5:**
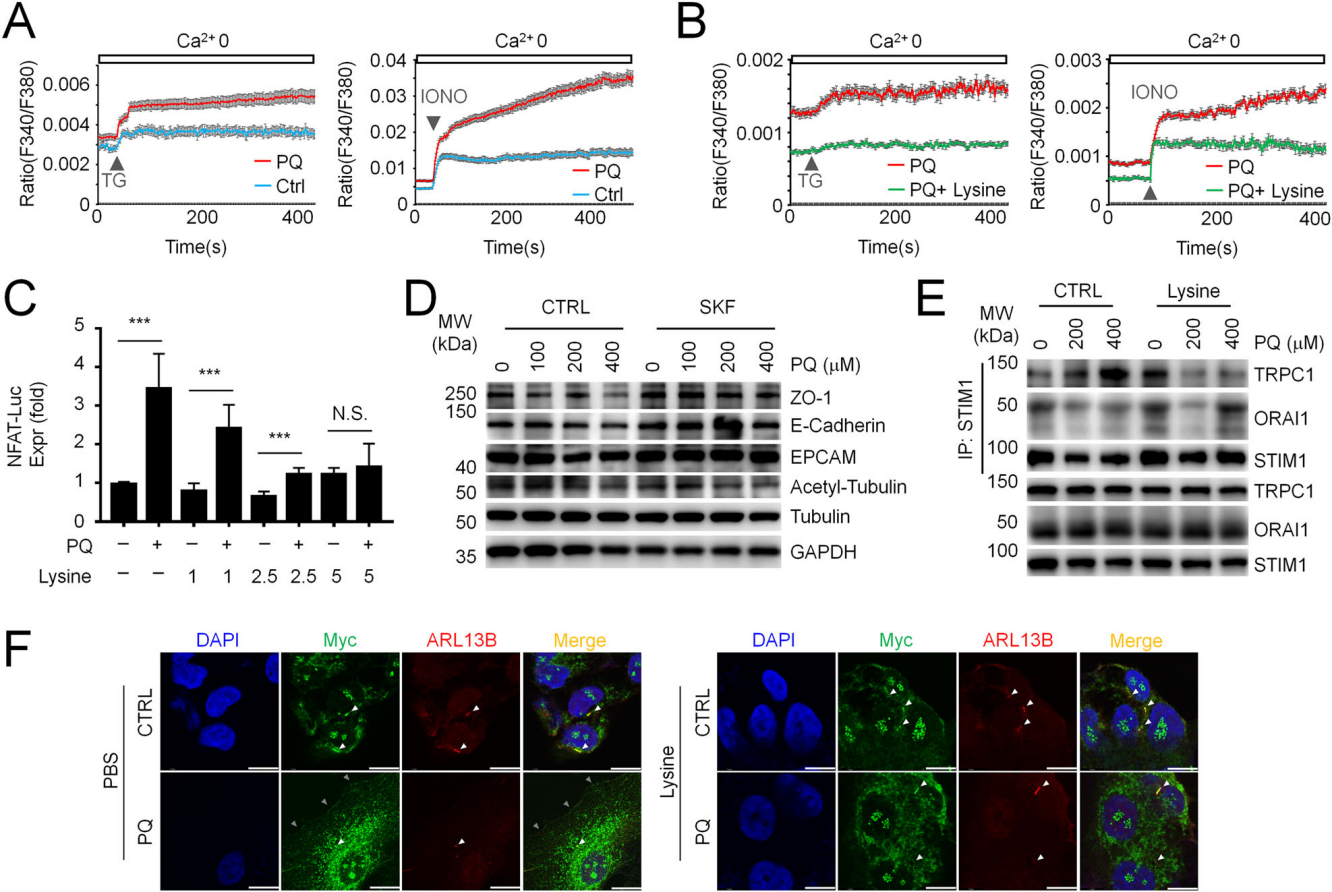
Lysine suppresses PQ-induced pathological STIM1-TRPC1 association and excessive intracellular calcium burden. Calcium image analysis in PQ-injured A549 cells compared to normal cells (**A**) and PQ-injured A549 cells w/wo lysine supplementation (**B**) with thapsigargin (TG) or ionomycin (IONO) stimulation. Mean ± SEM. **C** NFAT luciferase expression in A549 cells treated w/wo 800 μM PQ, together with 0-, 1-, 2.5-, or 5-fold lysine as in the culture medium as indicated, for 24 h. Mean ± SD, ***P < 0.001; Two-Way ANOVA. *n* = 3. NS not significant. **D** WB analysis of E-Cadherin, ZO-1, EPCAM and acetyl-α-Tubulin in PQ-injured A549 cells treated w/wo 5 μM SKF-96365 (SKF). **E** Co-IP analysis of STIM1 association with ORAI1 or TRPC1 in PQ-injured A549 cells with indicated concentration, together w/wo 5 mM lysine. **F** Immunofluorescence analysis of Myc and ARL13B in PQ-injured TRPC1-Myc stably expressed A549 cells w/wo lysine supplementation. Scale bar, 10 μm.

Next, we wondered how lysine-preserved cilia activation facilitated E-Cadherin integrity and epithelial property. Recent studies revealed that calcium signal activates a partial EMT process in epithelial cells, with largely diminished E-Cadherin but little alteration in the mesenchymal signature [34]. Consistently, our previous study identified that pathological STIM1-TRPC1 association-promoted calcium signals mediate PQ-induced EMT and toxicity in pulmonary epithelial cells, with E-Cadherin largely diminished [35]. Interestingly, cilia have been well recognized to mediate calcium influx [36]. These studies raised us to assume the potency of intracellular calcium homeostasis modulated by lysine preserved-cilia activation for alleviating ALI progression. We did observe increased calcium levels in whole cells and endoplasmic reticulum, the major intracellular calcium store, in injured pulmonary epithelial cells (Fig. 5A). Both of these calcium increasement could be normalized by lysine supplementation (Fig. 5B). Consistently, PQ-injured pulmonary epithelial cells present increased NFAT luciferase production, a well-established downstream of calcium signaling, which was inhibited by lysine supplementation in a dose dependent manner (Fig. 5C). Importantly, limitation of extracellular calcium entry by SKF-96365 (SKF) reversed PQ-reduced E-Cadherin and ZO-1 abundance, however, did not significantly modulate α-Tubulin acetylation (Figs. 5D and S2F). Moreover, lysine supplementation inhibited PQ-raised pathological association between STIM1 and TRPC1, with STIM1 binding to ORAI1 not dramatically affected (Figs. 5E and S2G). Immunofluorescence analysis further revealed that TRPC1 is present in multiple subcellular localizations, with signals observed in cilia as well. Interestingly, PQ injured-pulmonary epithelial cells present a much clearer plasma membrane (PM)-localized TRPC1, which was restored with lysine supplementation (Fig. 5F). These observations together suggested that lysine supplementation recovered the impairment of α-Tubulin acetylation for ciliogenesis, limited the abundance of PM-localized TRPC1, restrained extracellular calcium entry, and thus restored E-Cadherin integrity in injured pulmonary epithelial cells.

### Lysine replenishes acetyl-CoA for ciliogenesis to maintain calcium homeostasis and E-Cadherin integrity during ALI

**Fig. 6:**
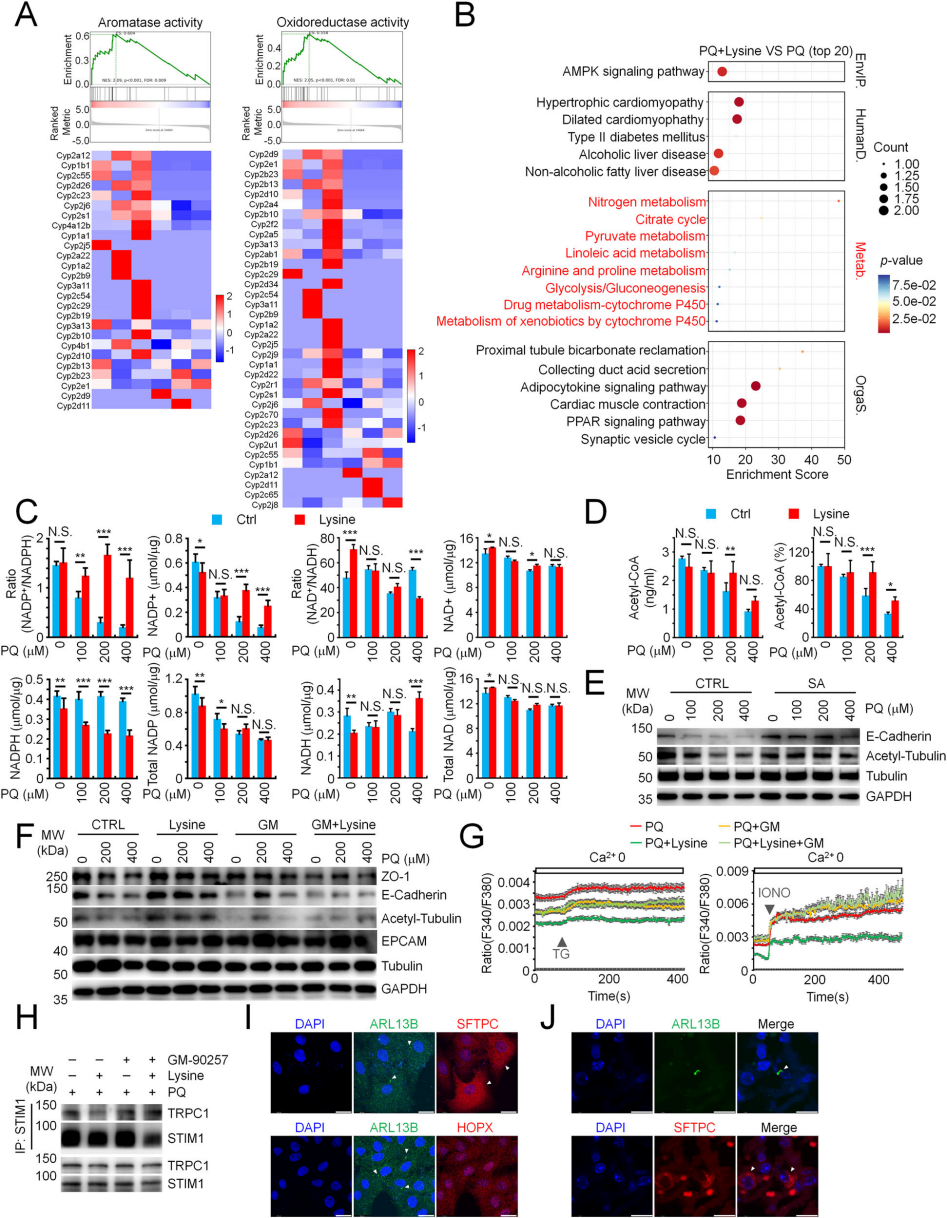
Lysine replenishes acetyl-CoA deficiency for restoration of ciliogenesis, calcium homeostasis, and E-Cadherin integrity in PQ-injured pulmonary epithelial cells. **A** GSEA analysis uncovered top-ranked molecular functions enhanced in PQ-injured lung tissues with lysine supplementation compared to the untreated group. **B** KEGG enrichment of the top 20 upregulated pathways in PQ-injured lung tissues with lysine supplementation compared to the untreated group. **C** Quantification of NADP and NADPH (left) or NAD and NADH (right) in PQ-injured A549 cells w/wo lysine supplementation. **D** ELISA analysis of acetyl coenzyme A (Acetyl-CoA) in PQ-injured A549 cells w/wo lysine supplementation. **E** WB analysis of E-Cadherin and acetyl-α-Tubulin in PQ-injured A549 cells treated w/wo 1 mM sodium acetate (SA). **F** WB analysis of ZO-1, E-Cadherin, acetyl-α-Tubulin and EPCAM in PQ-injured A549 cells w/wo lysine supplementation, together w/wo 0.5 μM GM-90257 (GM). **G** Calcium image analysis in PQ-injured A549 cells w/wo lysine supplementation, together w/wo 0.5 μM GM-90257 treatment. The cells were transiently stimulated with thapsigargin (TG) or ionomycin (IONO). Mean ± SEM. **H** Co-IP analysis of STIM1 association with TRPC1 in PQ-injured A549 cells w/wo lysine supplementation, together w/wo GM-90257 treatment. Immunofluorescence analysis of ARL13B, SFTPC, and HOPX in A549 cells (**I**) or normal lung tissues (**J**). Scale bar, 10 μm. Mean ± SD, *P < 0.05, **P < 0.01, ***P < 0.001; Two-Way ANOVA. NS not significant.

We then wondered how lysine reversed the deficiency in α-Tubulin acetylation and following ciliogenesis in injured pulmonary epithelial cells. To understand the potential molecular mechanism, we turned our sights to GSEA analysis of molecular functions and found that lysine supplementation significantly enhanced aromatase activity and oxidoreductase activity in lung tissues suffering from ALI. Overlapping genes of these two functions predominantly mediated NADPH oxidation to NADP+ (Fig. 6A), the coenzyme critical for multiple cellular metabolisms [37]. Consistently, KEGG analysis showed that metabolic pathways required NADP + /NADPH were enriched with lysine supplementation specifically in injured lung tissues but not in the physiological condition (Figs. 6B and S3). We further measured the abundance of NADP+ and NADPH in injured pulmonary epithelial cells and found a striking reduction of NADP+ and total NADP, while NADPH remained unaffected. Whereas lysine supplementation specifically restored the abundance of NADP+ and presented a reduction of NADPH in injured cells, with total NADP not much affected. In contrast, no much consistent difference was observed in NAD+ and NADH in lysine treated cells compared to the untreated group, yet lysine supplementation promoted a reduction of NAD+ and an increase of NADH in injured cells (Fig. 6C). These observations suggested a specific role of lysine facilitating the oxidization of NADPH to NADP+ during ALI, which raised the possibility of lysine in modulation of metabolic flux for α-Tubulin acetylation. Indeed, lysine has been reported to fuel acetyl-CoA biosynthesis, the known substrate for protein acetylation. Our analysis confirmed that lysine supplementation recovered the acetyl-CoA deficits in injured pulmonary epithelial cells (Fig. 6D). To test the role of acetyl-CoA for α-Tubulin acetylation in injured epithelial cells, we supplemented the injured cells with sodium acetate (SA), which mimicked lysine’s effects. The addition of sodium acetate reversed the deficiency of both E-Cadherin and α-Tubulin acetylation in injured pulmonary epithelial cells (Figs. 6E, and S4A). Moreover, GM-90257 (GM), the inhibitor of microtubule acetylation, abrogated the restoration of E-Cadherin abundance, α-Tubulin acetylation, and intracellular calcium burden by lysine supplementation (Figs. 6F, G, and S4B–E). Similarly, lysine supplementation-reduced pathological STIM1-TRPC1 association was also dampened by GM-90257 (Figs. 6H and S4F). These observations together suggested that lysine produced acetyl-CoA for the recovery of α-Tubulin acetylation and ciliogenesis in injured pulmonary epithelial cells. Notably, cilia-positive pulmonary epithelial cells present high expression of SFTPC, the marker of AT 2 cells (Fig. 6I). Moreover, some but not all subsets of SFTPC-positive cells present the cilia signal in lung tissues (Fig. 6J). Lysine thus would drive the regenerative activation in AT 2 subsets with the potency of ciliogenesis.

### Blood calcium levels are negatively correlated with the severity of ALI in patients

**Fig. 7:**
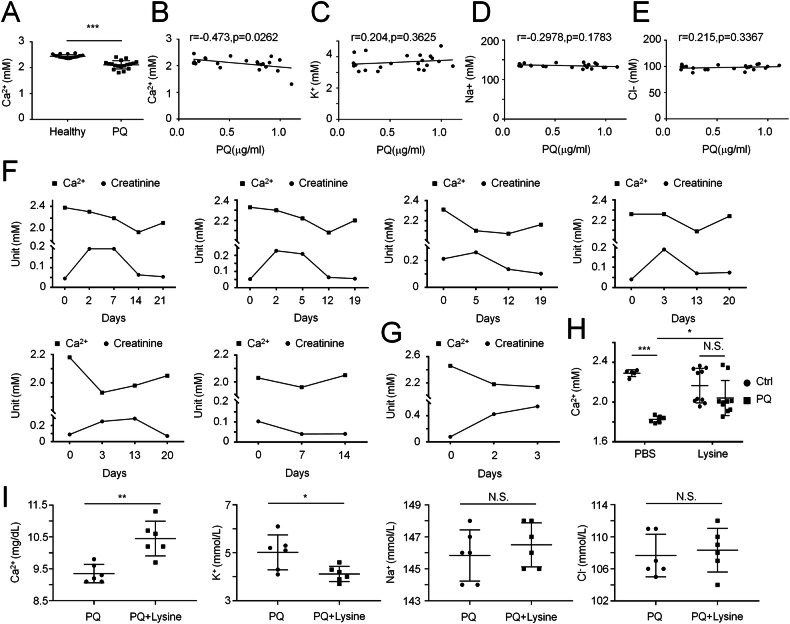
Serum calcium levels are negatively correlated with the severity of PQ poisoning-induced ALI progression. A retrospective analysis of the correlation between PQ and serum calcium (Ca^2+^) levels. **A** Mean ± SD, ***P < 0.001; unpaired two-tailed Student’s *t* test. *n* = 22 in healthy group, *n* = 18 in PQ group. **B** Pearson correlation coefficient. *n* = 22. The correlation between PQ and levels of serum potassium (K^+^, **C**), sodium (Na^+^, **D**) or chloride (Cl^-^, **E**). **F**, **G** Analysis of serum calcium levels (Ca^2+^) and serum creatinine levels in each PQ-poisoned patient during disease progression. **H** Levels of serum calcium levels (Ca^2+^) in PQ-poisoned mice treated w/wo lysine for 3 days. **I** Levels of serum calcium (Ca^2+^), potassium (K^+^), sodium (Na^+^) or chloride (Cl^-^) in PQ-poisoned cynomolgus monkey treated w/wo lysine for 3 days.

Lysine suppressed PQ-induced ALI by disrupting pathological STIM1-TRPC1 complex formation, thereby normalizing intracellular calcium overload. To validate calcium dysregulation as an important disease driver for ALI, we conducted a retrospective analysis of PQ-poisoned patients who suffered different degrees of ALI. By carefully analyzing the laboratory blood tests, we found that serum calcium levels were significantly decreased in PQ-poisoned patients compared to healthy volunteers. Further analysis revealed a negative correlation between the calcium levels and the severity of ALI, indicated by PQ concentrations in blood (Fig. 7A, B). In contrast, no significant correlation was observed in potassium, sodium, or chlorine (Fig. 7C–E). We further tracked the records of each patient with different severities of ALI. Serum calcium levels of patients who suffered moderate ALI were continuously reduced during the malignant progression, indicated by the increased blood creatinine levels, while restored, yet not fully, when patients recovered (Fig. 7F). Whereas, patients suffering fatal ALI died accompanied by extremely high creatinine levels and low blood calcium levels (Fig. 7G). We further analyzed serum calcium levels in two PQ-poisoned animal models and found that PQ-poisoned mice exhibited a significant reduction of serum calcium levels, which was dramatically restored by lysine treatment (Fig. 7H). Consistent with the observations in the mice model, lysine treatment also impressively alleviated the reduction of serum calcium levels in the PQ-poisoned cynomolgus model (Fig. 7I). Taken together, these multi-scale findings established calcium homeostasis as a non-redundant checkpoint in pulmonary resilience, with lysine-mediated STIM1-TRPC1 disassociation serving as a therapeutic linchpin.

## Discussion

Acute lung injury with concomitant pulmonary fibrosis is a progressive disease with high morbidity and early mortality among hospitalized patients. Anti-inflammatory strategies and antioxidants have been studied to treat or prevent ARDS progression, but have reached limited efficacy [7]. Moreover, though not all ARDS patients suffer PF progression, till now, antifibrotic strategies demonstrated minimal clinical benefit by identification of strategies to treat idiopathic pulmonary fibrosis (IPF), including bosentan, interferon gamma, etanercept, imatinib, everolimus, ambrisentan, macitentan, and warfarin [38–40]. Even approved IPF therapies like pirfenidone and nintedanib present limited efficacy on mortality [41–43]. These observations together underscore the urgent need for novel pharmacologic strategies targeting ALI and PF progression. Considering supportive therapy improves the prognosis of ARDS patients, endogenous regeneration dependent on AT 2 cell differentiation is critical for lung repair from injury. Others and our studies have shown that AT 2 cells either differentiate into AT 1 cells or present fibrotic potency during ALI [44, 45], and excessive fibrotic transition from persistent or severe alveolar epithelial damage, together with the following FMT promote PF progression. Here, we utilized the model of PQ poisoning-triggered severe alveolar epithelial damage to identify the importance of lysine-mediated subcellular compartmentalized modulation of intracellular calcium homeostasis in maintaining the pulmonary epithelial property during ALI. Previous investigations suggested that prolonged lysine administration (30 or 45 days) attenuates sepsis-induced lung injury in either cecal ligation puncture or LPS model [46, 47]. Given lysine’s documented anti-inflammatory efficacy against viral infections [24], its therapeutic benefit from prolonged treatment in sepsis likely stems from sustained immunomodulation. However, whether lysine confers protection in direct pulmonary damage-induced ALI within clinically relevant timeframes and relevant epithelial repair mechanisms distinct from broad anti-inflammation remained unexplored. By integrated analysis of PQ-poisoned murine/cynomolgus models and clinical cohorts of PQ-poisoned patients, we identified that lysine, a utilized clinical drug, as the potential adjunct strategy to treat ALI progression by restoration of ciliary activation that prevents pathological STIM1-TRPC1 association for excessive intracellular calcium influx in pulmonary alveolar epithelial cells, leading to a remarkable maintenance of impaired E-Cadherin integrity and epithelial property, further raising that strategies maintaining intracellular calcium homeostasis would be beneficial for treating ALI and PF, which requires further efforts to elucidate.

Recent studies revealed the importance of metabolic reprogramming in the modulation of AT 2 cells activation during ALI progression, with a common observation of impaired mitochondrial metabolism in pulmonary epithelial cells [16, 48]. The impairment of mitochondrial metabolism could be a stress response to cell injury, ascribing to either organelle dysfunction or functional exhaustion. We found an opposite activation between mitochondria and RNA metabolism during ALI. Previous studies have shown that metabolism rewire to glycolysis and energy crisis can be compensated by lipid metabolism during lung injury [16]. These observations together suggested a high possibility of functional exhaustion but not organelle dysfunction of mitochondria during ALI. Amino acid metabolism has been well characterized to support the energy crisis in the scenario of exhaustion in glucose and lipid metabolism; however, there is little understanding during ALI progression [21]. Our screening identifies the importance of lysine in replenishing acetyl-CoA for α-Tubulin acetylation, E-Cadherin integrity, and preservation of epithelial property in injured pulmonary epithelial cells, providing evidence for the metabolic compensation from amino acid metabolism. Consistently, NADP+ is accumulated, NADPH is consumed, and NAD+ is converted to NADH in injured pulmonary epithelial cells with lysine supplementation; the three processes occur during lysine metabolization to acetyl-CoA [49]. Notably, except lysine, other amino acids, like leucine, isoleucine, valine, threonine, tryptophan, and tyrosine, can also produce acetyl-CoA [21, 50], which are all reduced in the serum of PQ-poisoned patients. Therefore, further studies are required to examine the combination of these amino acids for preventing or alerting to the injury of pulmonary alveolar epithelial cells and ALI progression.

AT 2 cells are well characterized to differentiate into AT 1 cells during pulmonary injury for maintaining the function of pulmonary gas exchange [51]. A failure of AT 2 cells differentiation into AT 1 cells has been reported to develop fibrosis in mice [52]. Several signaling have been revealed to be required for AT 2 cell proliferation [53], however, it is remained elusive for AT 2-to-AT 1 differentiation. WNT, Notch, BMP, and TGFβ have all been reported to participate in AT 2-to-AT 1 differentiation, while the signaling networks inside cells are not well elucidated [53]. Calcium ions, important second messengers that exhibit almost omnipotent functions, have been well recognized to participate in the above signaling cascades [54–57]. Our results revealed the importance of intracellular calcium homeostasis in potentially maintaining AT 2 cell fates, especially the E-Cadherin integrity, while excessive calcium burden facilitates the partial EMT process. These studies thus emphasized the appropriate and dynamic activation of calcium signaling to maintain the stemness of AT 2 to differentiate into AT 1. Note, intracellular calcium signaling would be dynamically modulated by fluxes from both extracellular calcium pools and calcium stores inside cells [57], both of which are increased during ALI progression and can be normalized by lysine supplementation and ciliary TRPC1 subcellular localization. As cilia are observed in specific AT 2 subsets, further efforts are thus required to clarify the precise modulation of intracellular calcium signaling for maintaining the stemness of AT 2 subsets.

In conclusion, our previous study found that PQ targets the STIM1-TRPC1 axis for extracellular calcium entry, followed by intracellular Ca^2+^ overload in pulmonary epithelial cells and thus results in the damage of alveolar epithelial cells [35]. Here, we identified lysine as an adjunct strategy for PQ-induced ALI progression by replenishing acetyl-CoA to restore injured ciliary activation and intracellular calcium homeostasis, from which we further emphasized the potency of calcium signals for modulation of AT 2 cells differentiation and activation, providing potential molecular targets and safe strategies for treating ALI progression.

## Materials and methods

### Cell culture

A549 cells were cultured in DMEM Medium (BasalMedia, L110KJ) supplemented with 10% fetal bovine serum (Excell, FSP500), penicillin (100 IU/ml, Genom, GNM15140) and streptomycin (100 μg/ml, Genom, GNM15140). MLE-12 cells were cultured in Dulbecco’s Modified Eagle Medium/Nutrient Mixture F-12 (DMEM/F12, AWcell-0005) supplemented with 2% fetal bovine serum (GIBCO, 42Q1095K), 1% GlutaMAX-1 (AWcell-0900), 10 mM HEPES buffer solution (GIBCO, 15630-080), 1% ITS (AWcell-4507), 10 nM hydrocortisone (AWcell-8450), 10 nM β-estradiol (AWcell-8140), penicillin (100 IU/ml, Genom, GNM15140) and streptomycin (100 μg/ml, Genom, GNM15140). These cell lines were authenticated and grown at 37 °C in a 5% carbon dioxide incubator and were passaged following trypsinization. The cells were starved with FBS-free culture medium for 12 h before each experiment.

### Mice

Six-week-old male C57BL/6 mice were randomly and evenly divided into groups as indicated in specific experiments. Group assignment and data analysis were performed by different researchers in a blinded manner. The number of mice in each experiment is shown in the figure legend. The mice were intraperitoneally injected with PQ (50 mg/kg, Sigma-Aldrich, 36541) for one time, whereas vehicle control or lysine (10 mg/kg, Sangon Biotech, A602769) was intraperitoneally injected every day for 3 days, or as indicated. The daily food and water uptake of the treated mice was recorded every day. Mice were sacrificed on day 4 or as indicated, and lung lobes were removed, fixed in 4% paraformaldehyde, dehydrated, and subjected to paraffin embedding. Lung microsections (5 μm) were stained with hematoxylin & eosin (H&E), and Masson’s trichrome to visualize fibrotic lesions, or examined by immunofluorescence using Anti-ZO-1 antibody (Cell Signaling Technology, 13663, 1:200). Cheek blood samples were prepared, and the serum was isolated by centrifugation at 2000 rpm for 5 min. The serum was monitored in Servicebio (Wuhan, China) for serum calcium levels. All the animal studies were approved by the Ethics Committee of Shanghai General Hospital and all methods were performed in accordance with the relevant guidelines and regulations, and the IACUC Protocol number was 2022AWS0108.

### PQ-poisoned cynomolgus model

A paraquat-induced injury model was developed in two cynomolgus monkeys (Medicilon Inc., Shanghai, China) and evaluated at serial time points. Briefly, five-year-old male cynomolgus monkeys were orally administered 12.5 mg/kg PQ once, mainly determined by body surface area (BSA)-based allometric scaling. The formula applied was: Monkey Dose (mg/kg) = Mouse Dose (mg/kg) × Mouse Weight (g) × 64.1/Monkey Weight (g). PQ-poisoned cynomolgus monkeys were further treated with vehicle or 40 mg/kg lysine by intravenous injection every day for three days. Blood was collected by femoral venipuncture as indicated. The population of immune cells was measured by laboratory blood tests, and the serum calcium levels were monitored by serum biochemistry. For CRP analysis, serum was prepared by centrifugation of collected blood at 3500 rpm for 10 min at room temperature. The prepared serum was then subjected to CRP ELISA analysis according to the manufacturer’s instructions (Life Diagnostics, Inc., CRP-3). All methods were performed in accordance with the relevant guidelines and regulations. The IACUC Protocol number for this study was 19185-20001.

### Hydroxyproline assay

To estimate the collagen synthesis in the lungs, we measured the amount of hydroxyproline via ELISA analysis (Shanghai Yubo Biological Technology). Briefly, lung tissues were homogenized in 1 ml phosphate-buffered saline (PBS) and centrifuged at 4000 rpm for 15 min. The supernatant was collected, and BCA measurement was performed to examine the total amount of proteins in lung tissues. Hydroxyproline content was measured according to the Manufacturer’s instructions and determined by a colorimetric assay using a spectrophotometer at a wavelength of 450 nm (Thermofisher, Vantaa, Finland). Data were presented as μg hydroxyproline/μg of lung tissue. The number of samples is shown in the figure.

### Clinical samples

We retrospectively reviewed a part of patients with paraquat poisoning (*n* = 18) at Qilu Hospital of Shandong University and normal healthy volunteers (*n* = 22) at Shanghai General Hospital. Informed consent was obtained from all individual participants included in the study. Overall, the biochemical analysis of electrolytes and the serum creatinine levels previously recorded were analyzed. The concentrations of PQ in serum were measured by High-Performance Liquid Chromatography. All the studies were approved by the Ethics Committee of Shanghai General Hospital and Qilu Hospital. The clinical information of PQ-poisoned patients was collected by one researcher and analyzed by another researcher with blinding.

### Quantitative RT-PCR

A549 cells were treated with PQ in a dose-dependent manner, as indicated, for 12 h. The PQ-containing medium was then removed, and the treated cells were further cultured in fresh medium with or without 5 mM lysine for 18 h. The cells were lysed in TRIZOL (Vazyme Biotech Co, R701-02), and total RNA was extracted and reverse transcribed into cDNA by HiScript III RT SuperMix kit (Vazyme Biotech Co, R323). The mRNA expression of indicated genes was determined by semi-quantification with ChamQ SYBR Color qPCR Master Mix (Vazyme Biotech Co, R323, Q411-02) in QuantStudio 7 Flex. Data were normalized to Actin and calculated by 2^[− (Ct target gene−Ct Actin)]^. The sequence of the PCR primers was listed in Supplementary Table 1 [35]. The experiment included at least three independent biological replicates.

### Western blot

Cells were treated as those in quantitative RT-PCR, and protein lysates were separated by 10% SDS-PAGE gels and transferred onto a nitrocellulose filter membrane (Pall, 66485), blocked with 5% non-fat milk in phosphate-buffered solution with 0.1% Tween 20 (PBST). Primary antibodies were incubated at 4 °C overnight, including Anti-STIM1 (Cell Signaling Technology, 5668, 1:3000), Anti-ORAI1 (Santa Cruz, sc-377281, 1:500), Anti-TRPC1 (Proteintech, 19482-1-AP, 1:2000), Anti-E-Cadherin (Proteintech, 60335-1-Ig, 1:2000), Anti-ZO-1 (Cell Signaling Technology, 13663, 1:3000), Anti-EPCAM (Proteintech, 21050-1-AP, 1:2000), Anti-Vimentin (Cell Signaling Technology, 5741S, 3:1000), Anti-N-Cadherin (Proteintech, 66219-1-Ig, 1:2000), Anti-α-SMA (Abcam, ab7817, 1:3000), Anti-α-Tubulin (Proteintech, 11224-1-AP, 1:2000), Anti-acetyl-α-Tubulin (Proteintech, 66200-1-Ig, 1:2000), and Anti-GAPDH (Proteintech, 60004-1-1 g, 1:10000). The experiment included a minimum of two independent biological replicates. Uncropped Blots could be found in Supplemental Material-Uncropped Blots.

### Immunoprecipitation

A549 cells were treated with 200 or 400 μM PQ for 12 h. The PQ-containing medium was then removed, and the treated cells were further cultured in fresh medium with or without 5 mM lysine for 18 h and lysed in RIPA buffer (Beyotime, P0013D) with the addition of proteasome inhibitor (Roche, 04693124001). The lysates were quantified by the Bicinchoninic acid (BCA) method and incubated with STIM1 antibody (Cell Signaling Technology, 5668, 1 μl/sample) overnight, followed by incubation with 20 μl protein A/G beads (Proteintech, PR40025) for another 3 h at 4 °C. The immune complexes were washed three times with PBS and subjected to Western Blot. Specific antibodies were used for STIM1 (Cell Signaling Technology, 5668, 1:3000), ORAI1 (Santa Cruz, sc-377281, 1:500), and TRPC1 (Proteintech, 19482, 1:2000).

### Immunofluorescence

A549 cells were plated on glass coverslips in the 24-well plate. After attachment, the cells were treated with 100, 200 or 400 μM PQ for 12 h. The PQ-containing medium was then removed, and the treated cells were further cultured in fresh medium with or without 5 mM lysine for 18 h. The treated cells were then washed with PBS and fixed in 4% paraformaldehyde, permeabilized with PBS containing 0.1% Triton-X-100, blocked with PBS containing 0.2% BSA (w/v), incubated with the indicated primary antibodies at 4 °C overnight and secondary antibodies at R.T. for 1 h and imaged by a laser scanning confocal microscope (Leica TCS SP8, Mannheim, Germany). Primary antibodies utilized were anti-ARL13B (Proteintech, 17711-1-AP, 1:200), anti-ARL13B (Proteintech, 66739-1-Ig, 1:200), anti-ZO-1 (Cell Signaling Technology, 13663, 1:200), anti-Myc tag (Abmart, M20002, 1:500), anti-SFTPC (Abcam, ab90716, 1:200), and HOPX (Proteintech, 11419-1-AP, 1:200). The secondary antibodies utilized were 488-conjugated goat anti-mouse secondary antibody (Thermo, A32723, 1:2000) and 594-conjugated goat anti-rabbit secondary antibody (Thermo, A32740, 1:2000), or 594-conjugated goat anti-mouse secondary antibody (YaJi Biological, bs-0368G-AF594, 1:1000) and 488-conjugated goat anti-rabbit secondary antibody (YaJi Biological, bs-0259G-AF488, 1:1000). The experiment included at least three independent biological replicates.

### Acetyl-CoA measurement

A549 cells were plated in the 12-well plate at a concentration of 2 **×** 10^5^/well. After attachment, the cells were treated with 100, 200 or 400 μM PQ, as indicated, for 12 h. The PQ-containing medium was then removed, and the treated cells were further cultured in fresh medium with or without 5 mM lysine for 18 h. The cells were then lysed in 200 μl PBS with repeated freeze-thaw cycles, and the concentration of acetyl-CoA was measured by using an Acetyl Coenzyme A ELISA Kit (Sangon Biotech, D751001, Shanghai) according to the manufacturer’s instructions. The experiment included two independent biological replicates.

### NAD/NADP measurement

For NADP measurement, A549 cells were plated in a 24-well plate. For NAD measurement, A549 cells were plated in a 12-well plate. After attachment, the cells were treated with 100, 200 or 400 μM PQ, as indicated, for 12 h. The PQ-containing medium was then removed, and the treated cells were further cultured in fresh medium with or without 5 mM lysine for 18 h. The cells were then lysed in the indicated lysis buffer and examined by using a NAD + /NADH Assay Kit (Beyotime, S0175, Shanghai) and NADP + /NADPH Assay Kit, respectively (Beyotime, S0179, Shanghai) according to the manufacturer’s instructions. The experiment included two independent biological replicates.

### Reporter gene assay

A549 cells were transfected with NFAT-luciferase/Renilla plasmids by using polyjet transfection reagent (Signagen, SL100688). The transfected cells were pretreated w/wo lysine in a dose-dependent manner for 2 h, followed by 800 μM PQ stimulation for an additional 24 h. The amount of NFAT-luc or Renilla was measured by using the dual-luciferase Kit Assay Reporter System (Vazyme Biotech Co, DL101-01) and examined by using a luminometer (Varioskan Flash, Thermo Fisher, MA, USA). The data were presented as the ratio of the NFAT-luc luminescence to Renilla luminescence. The experiment included two independent biological replicates.

### Single-cell Ca^2+^ measurements

A549 cells were plated in 35 mm glass-bottom dishes and treated with 400 μM PQ for 12 h. The PQ-containing medium was then removed, and cells were further cultured in fresh medium with or without 5 mM lysine for 18 h. The treated cells were then incubated with 2 μM Fura-2 AM (Invitrogen™, F1221) diluted in Ca^2+^ free D-Hanks buffer for 30 min. The stained cells were stimulated with 1 μM ionomycin or thapsigargin, as indicated, for measurements of calcium fluxes in a THUNDER Imager DMi8 microscope (Leica, Mannheim, Germany). Fura-2 (340/380) filter set (pE-340fura, CoolLED, UK), a 20 × 0.8 NA. An objective lens and a K8 camera were used to capture images at a frequency of 1 image pair every 2 s. Relative fluorescence ratio at wavelengths of 340 nM and 380 nM (F340/F380) was measured and utilized for the assessment of cytoplasmic calcium levels. The experiment included two independent biological replicates.

### GC-MS metabolomics analysis

Human blood from PQ poisoning patients was obtained at Qilu Hospital of Shandong University. Plasma was isolated by Ficoll density centrifugation and collected to carry out the GC-MS Metabolomics Analysis (Oebiotech, Shanghai).

### RNA-Seq

30 mg lung tissues from 50 mg/kg PQ-poisoned mice model, treated with or without 10 mg/kg lysine, were isolated and subjected to RNA-seq analysis at Oebiotech, Shanghai.

### Statistical analysis

Data are represented as mean ± SD or mean ± SEM as indicated. The comparisons between any two groups were analyzed by a two-tailed one-sample Student’s *t* test, or in an experiment with multiple comparisons, were performed using one-way or two-way ANOVA followed by Bonferroni post-tests, as indicated. Pearson correlation coefficient was performed to analyze the correlation between electrolytes and PQ in blood. Kaplan-Meier analysis was performed to analyze the survival rate after PQ poisoning. *P < 0.05, **P < 0.01, ***P < 0.001.

## Supplementary information

Supplementary figures and table

## Data Availability

The data of this study are available from the corresponding author upon reasonable request. Some data may not be made available because of privacy or ethical restrictions.
