# Supplementary material for: Lysine attenuates acute lung injury by restoring α-tubulin acetylation and ciliary activity
Source: Cell Death Discov. 2026 Mar 16;12:150. doi: 10.1038/s41420-026-03025-x (PMC13039107; doi:10.1038/s41420-026-03025-x)

**Supplemental Figure Legends**

**Figure S1. Lysine alleviates PQ-induced ALI progression. (A-B)** Daily food intake (A) and daily water intake (B) were recorded in control (Ctrl) or PQ-injured mice treated w/wo lysine for 3 days. Mean ± SD, *P < 0.05, **P < 0.01, ***P < 0.001; Two-way ANOVA. **(C)** Daily weight change of PQ-injured mice treated w/wo lysine for 3 days. Mean ± SD, *P < 0.05; Two-Way ANOVA. n = 5 in each group. **(D)** Representative images of lung tissue from control (Ctrl) or PQ-injured mice treated w/wo lysine for 3 days. **(E-F)** Abundance of gene expression relevant to AT 1 cells (E) or AT 2 cells (F) by RNA-seq analysis.

**Figure S2. Semi-quantification analysis of WB results related to Fig. 4 and Fig. 5, and WB analysis of E-Cadherin and Vimentin expression in PQ-injured MLE12 cells w/wo lysine supplementation (B).**

**Figure S3. KEGG enrichment of top 20 upregulated pathways in Lysine-treated lung tissues compared to untreated group.**

**Figure S4. Semi-quantification analysis of WB results related to Fig. 6.**

**
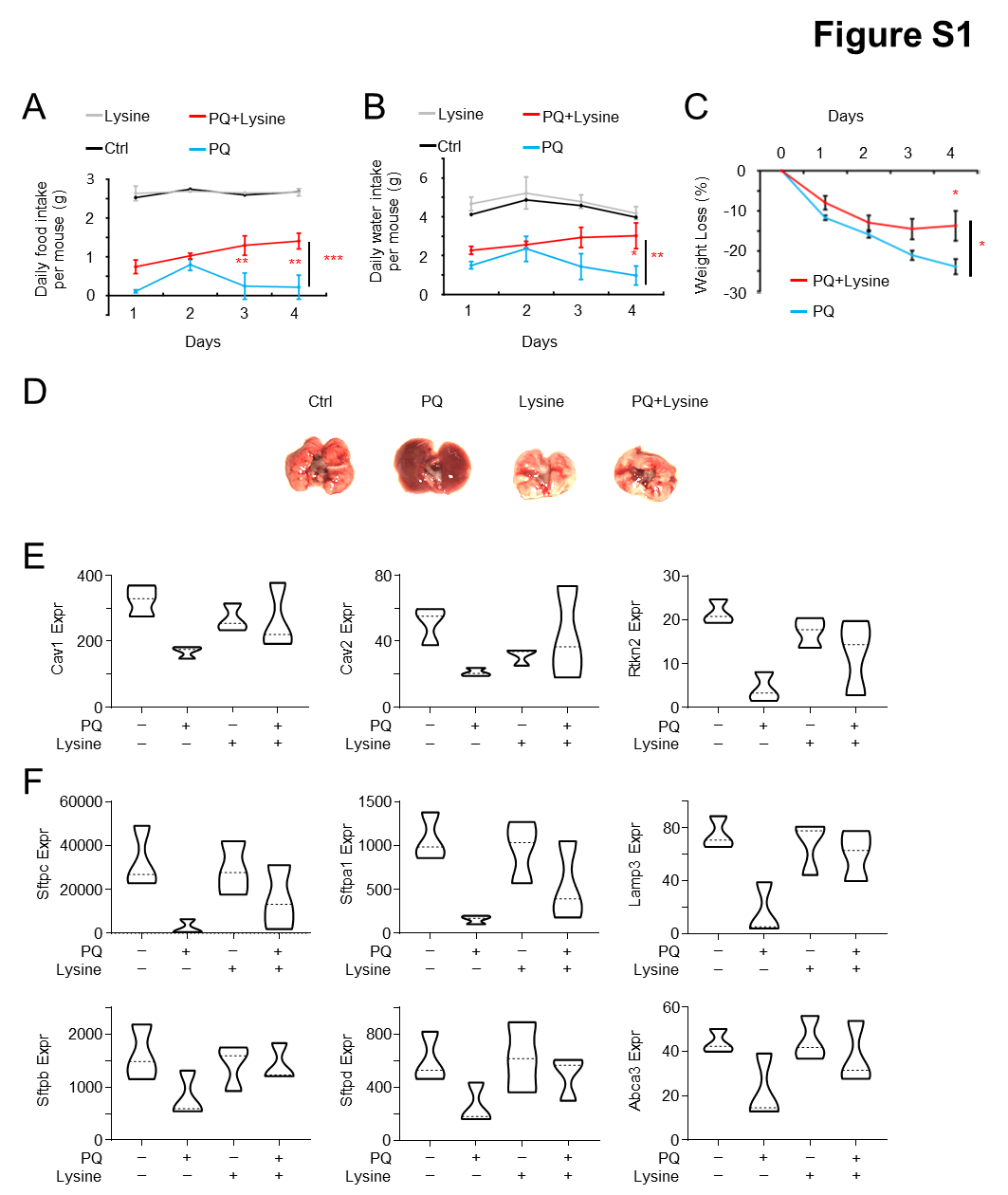
**

**
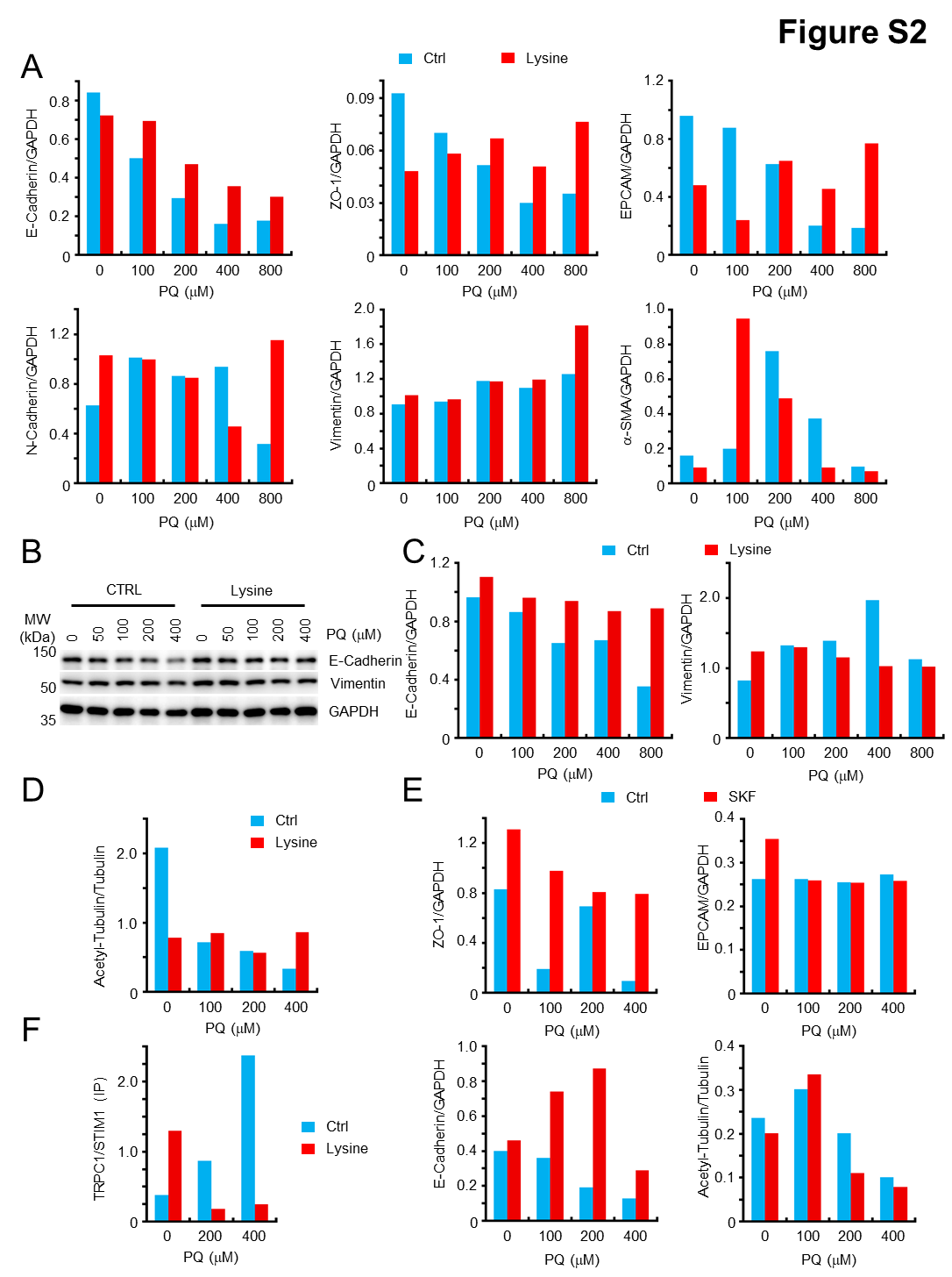
**


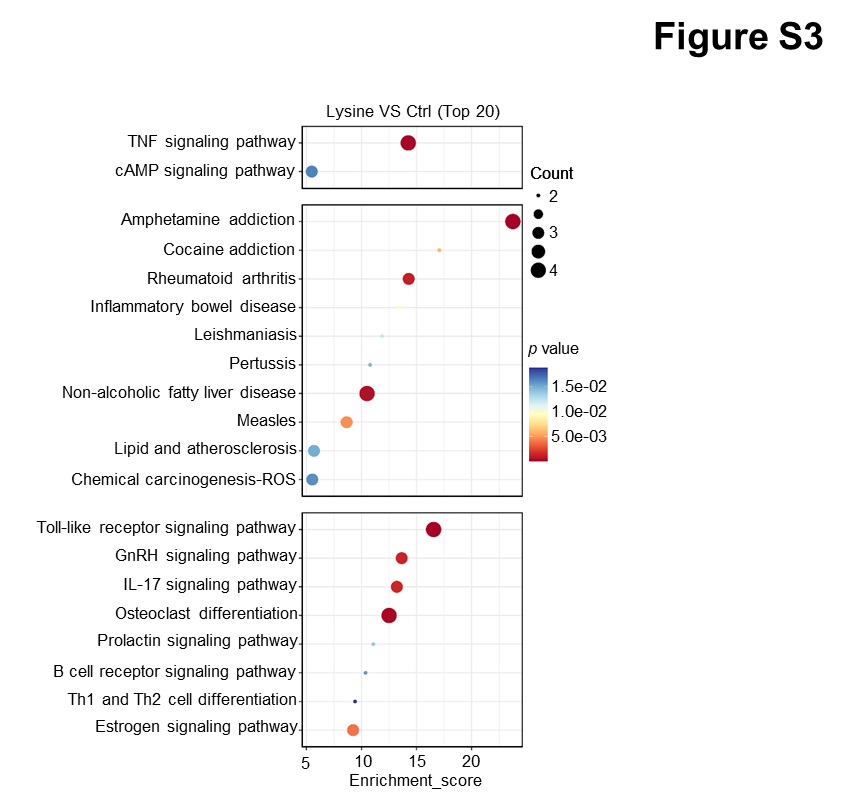


**
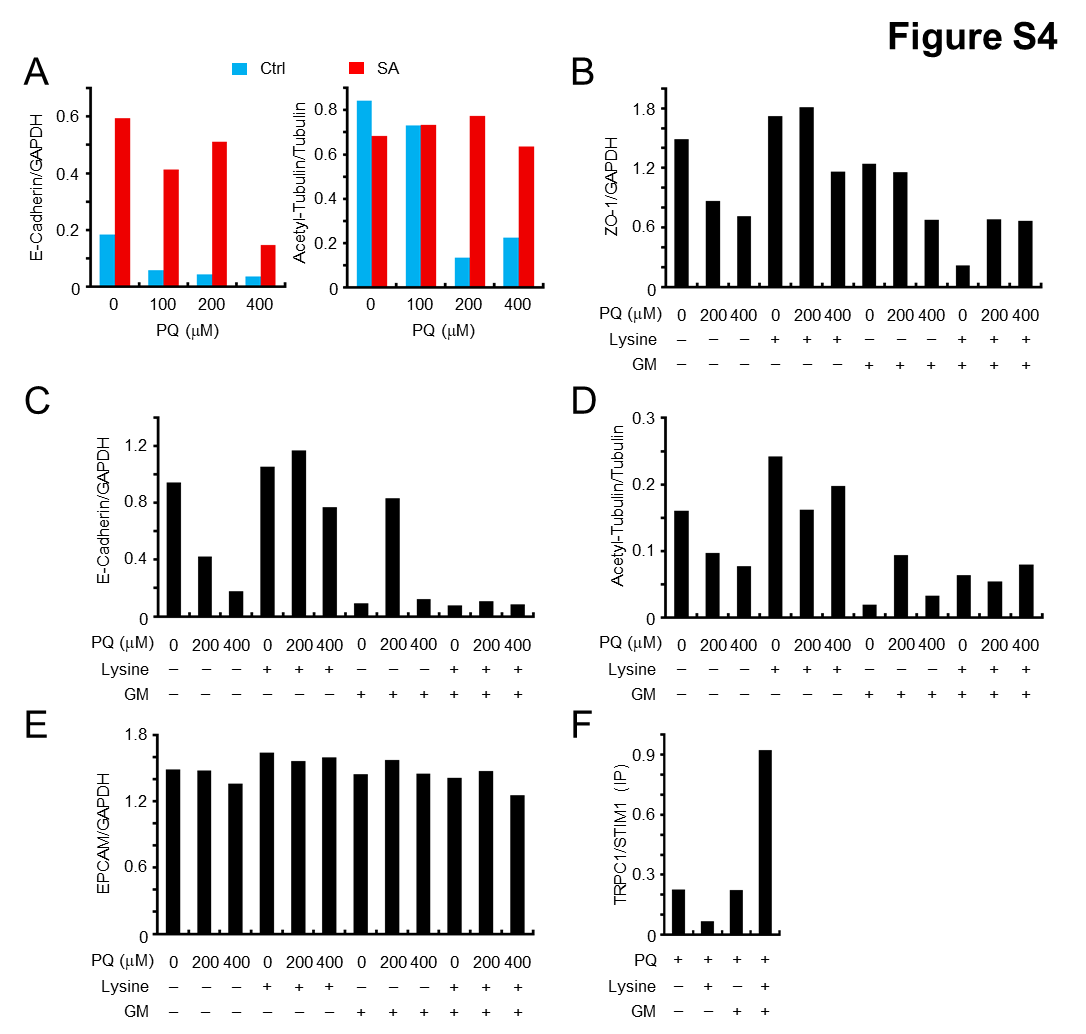
**

Table S1. PCR primers

| Gene Sequence | | | |
| --- | --- | --- | --- |
| *Gapdp* | sense  antisense | 5’ ACCCAGAAGACTGTGGATGG 3’  5’ TTCAGCTCAGGGATGACCTT 3’ |  |
| *E-Cadherin* | sense  antisense | 5’ TTCCTCCCAATACATCTCCC 3’  5’ TTGATTTTGTAGTCACCCACC 3’ |  |
| *Vimentin* | sense  antisense | 5’ CTCTTCCAAACTTTTCCTCCC 3’  5’ AGTTTCGTTGATAACCTGTCC 3’ |  |
| *IL-1b* | sense  antisense | 5’ AGCTACGAATCTCCGACCAC 3’  5’ CGTTATCCCATGTGTCGAAGAA 3’ |  |
| *TNF* | sense  antisense | 5’ CCTCTCTCTAATCAGCCCTCTG 3’  5’ GAGGACCTGGGAGTAGATGAG 3’ |  |
| *IL-6* | sense  antisense | 5’ ACTCACCTCTTCAGAACGAATTG 3’  5’ CCATCTTTGGAAGGTTCAGGTTG 3’ |  |
| *Ccl2* | sense  antisense | 5’ CAGCCAGATGCAATCAATGCC 3’  5’ TGGAATCCTGAACCCACTTCT 3’ |  |
| *Cxcl5* | sense  antisense | 5’ AGCTGCGTTGCGTTTGTTTAC 3’  5’ TGGCGAACACTTGCAGATTAC 3’ |  |


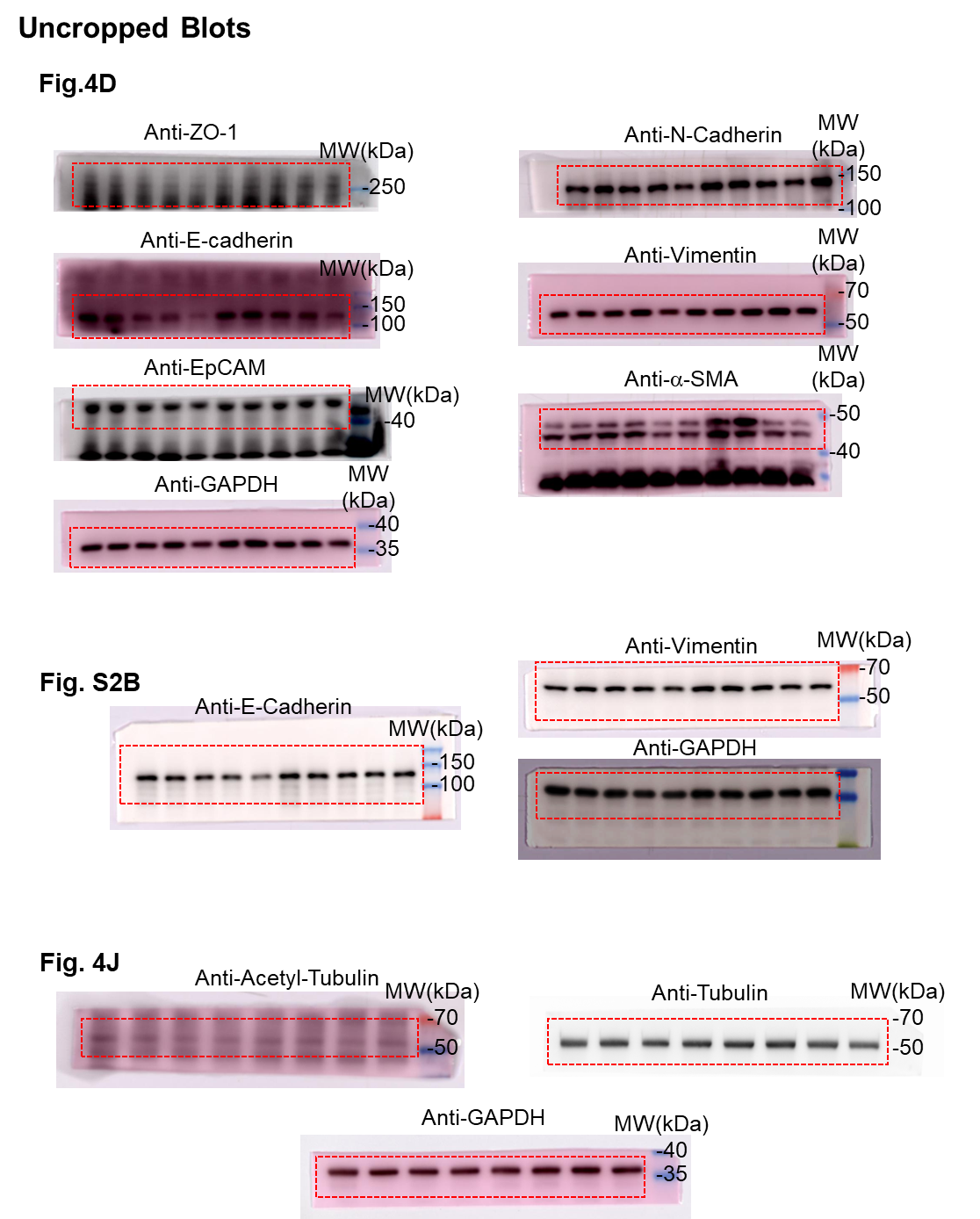


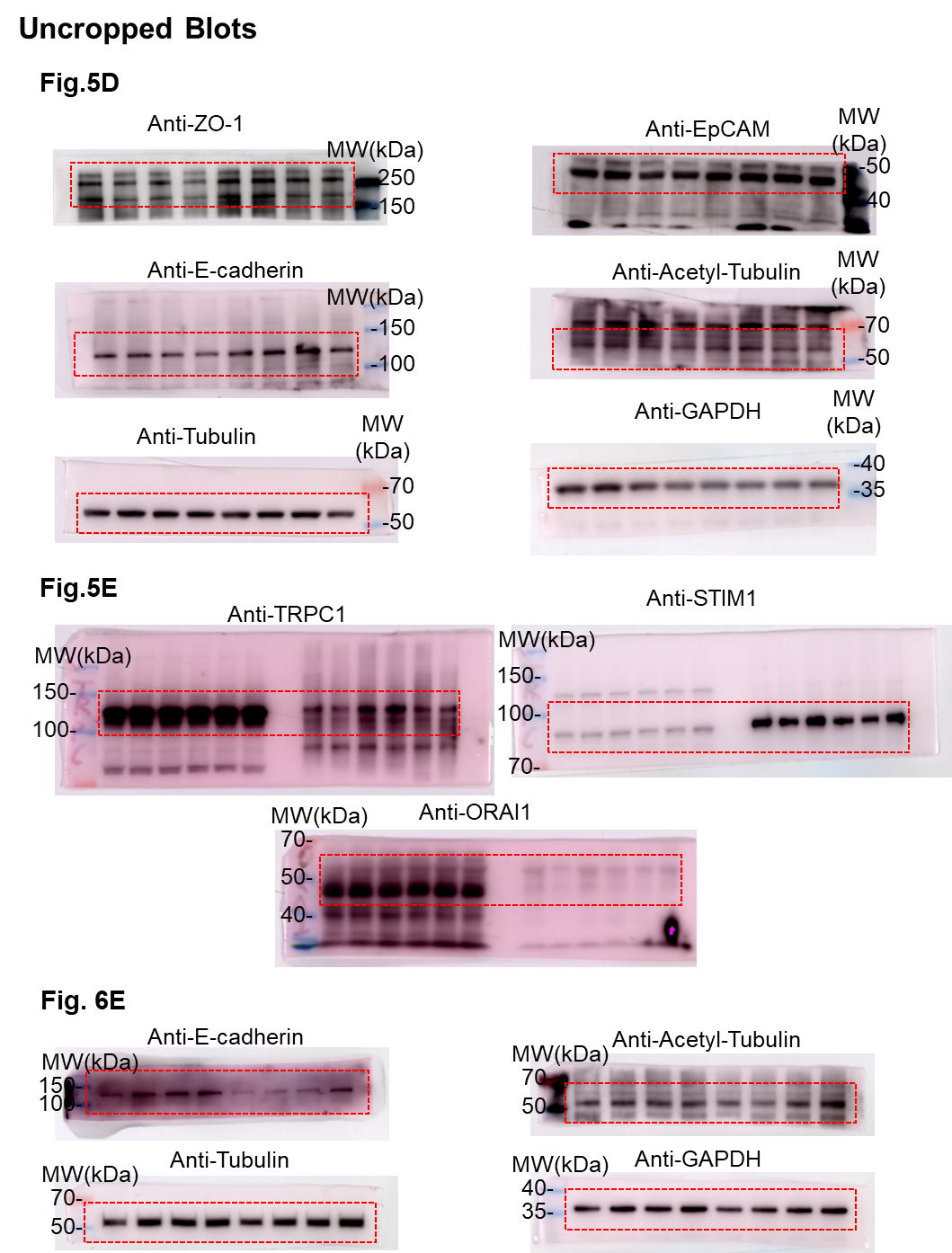


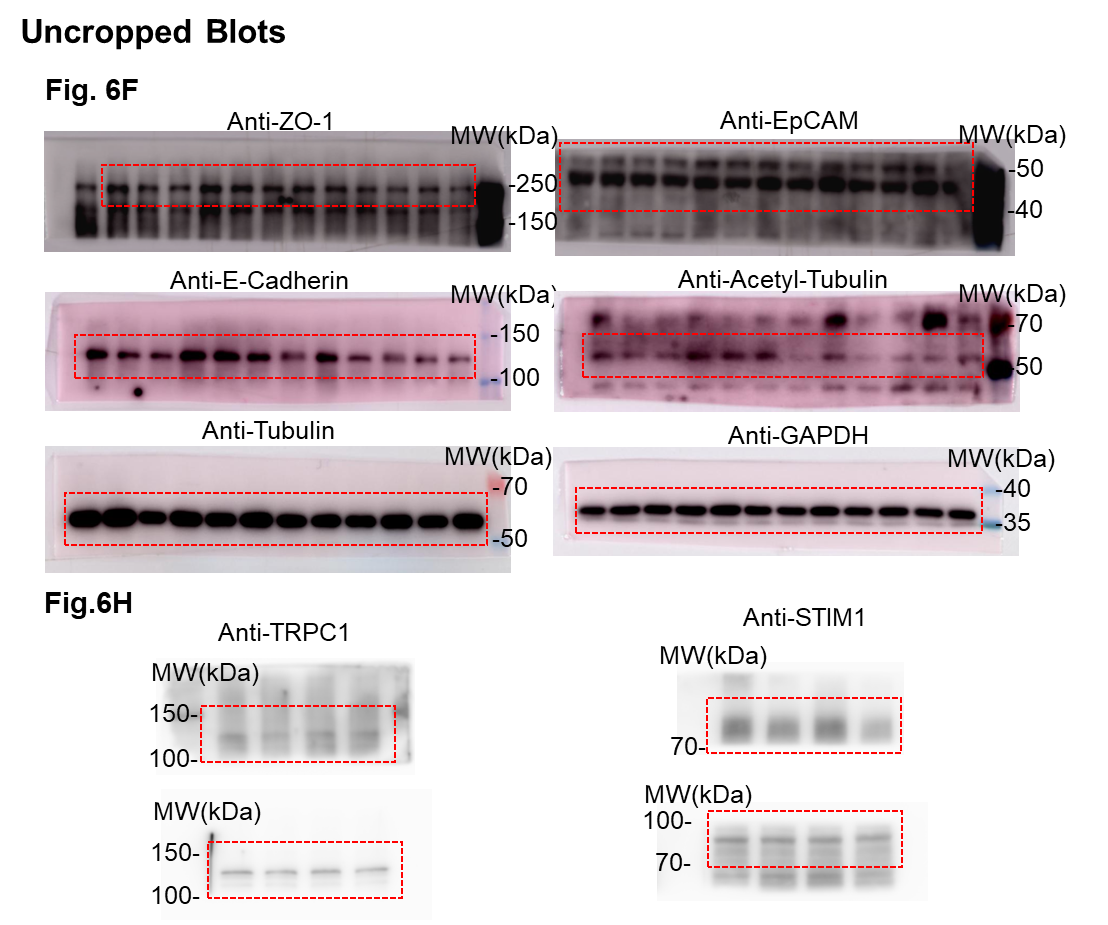

Supplement: Supplementary file 1 — Supplementary figures and table [file 41420_2026_3025_MOESM1_ESM.docx]
